# Expiratory flow rate, breath hold and anatomic dead space influence electronic nose ability to detect lung cancer

**DOI:** 10.1186/1471-2466-14-202

**Published:** 2014-12-16

**Authors:** Andras Bikov, Marton Hernadi, Beata Zita Korosi, Laszlo Kunos, Gabriella Zsamboki, Zoltan Sutto, Adam Domonkos Tarnoki, David Laszlo Tarnoki, Gyorgy Losonczy, Ildiko Horvath

**Affiliations:** Department of Pulmonology, Semmelweis University, 1/C Dios arok, Budapest, 1125 Hungary; Department of Pediatrics, Heim Pal Children’s Hospital, 86 Ulloi street, Budapest, 1082 Hungary; Department of Radiology and Oncotherapy, Semmelweis University, 78/a Ulloi street, Budapest, 1082 Hungary

**Keywords:** Biomarkers, Breath test, Electronic nose, Lung cancer, Volatile organic compounds

## Abstract

**Background:**

Electronic noses are composites of nanosensor arrays. Numerous studies showed their potential to detect lung cancer from breath samples by analysing exhaled volatile compound pattern (“breathprint”). Expiratory flow rate, breath hold and inclusion of anatomic dead space may influence the exhaled levels of some volatile compounds; however it has not been fully addressed how these factors affect electronic nose data. Therefore, the aim of the study was to investigate these effects.

**Methods:**

37 healthy subjects (44 ± 14 years) and 27 patients with lung cancer (60 ± 10 years) participated in the study. After deep inhalation through a volatile organic compound filter, subjects exhaled at two different flow rates (50 ml/sec and 75 ml/sec) into Teflon-coated bags. The effect of breath hold was analysed after 10 seconds of deep inhalation. We also studied the effect of anatomic dead space by excluding this fraction and comparing alveolar air to mixed (alveolar + anatomic dead space) air samples. Exhaled air samples were processed with Cyranose 320 electronic nose.

**Results:**

Expiratory flow rate, breath hold and the inclusion of anatomic dead space significantly altered “breathprints” in healthy individuals (p < 0.05), but not in lung cancer (p > 0.05). These factors also influenced the discrimination ability of the electronic nose to detect lung cancer significantly.

**Conclusions:**

We have shown that expiratory flow, breath hold and dead space influence exhaled volatile compound pattern assessed with electronic nose. These findings suggest critical methodological recommendations to standardise sample collections for electronic nose measurements.

## Background

Exhaled breath contains thousands of volatile molecules and their levels change with cellular metabolism and oxidative stress [1]. Therefore, it is not surprising that altered exhaled volatile compound levels were found in various disorders of the respiratory system such as lung cancer [2–9], malignant mesothelioma [10, 11], obstructive airway diseases [12–14], sarcoidosis [15], obstructive sleep apnoea [16–18], respiratory infections [19, 20] and in patients with lung transplantation [21].

Breath tests have unique advantages that they are completely non-invasive, well-tolerable, hold no risks for side effects and can be performed even in very sick patients; therefore they pose an ideal tool for disease screening. This fact is particularly important in lung cancer, as early diagnosis is associated with significantly better prognosis [22].

Gas-chromatography mass-spectometry (GC-MS) is the gold standard for exhaled volatile compound measurement. However, these machines are very expensive and need special skills and experience. Electronic noses, composites of nanosensor arrays and in-built processors, represent another, generally cheaper and easier technique to measure exhaled volatile compounds [5]. These devices cannot identify and quantify the molecules in gas mixtures but are able to compare and discriminate gaseous samples based on the contour of volatile substances (“breathprint”). In line with this, electronic noses could distinguish exhaled breath samples of patients with different respiratory disorders from those of healthy subjects [23–25].

Lung cancer is probably the most frequently investigated respiratory disorder in electronic nose research. Numerous studies demonstrated that various electronic noses could identify lung cancer by analysing exhaled breath samples [5–9]. Cyranose 320 is one of the most widely used electronic noses and it applies a carbon black conducting polymer sensor array which is selective for polar compounds and its lower detection limit is around 0.1 ppm (particles per million) [26]. Numerous volatile substances were identified as being altered in lung cancer in this concentration range, such as acetone [3, 4, 27, 28], isoprene [3, 4, 27, 29], benzene [2], xylene [3], pentane [30], ethanol [28] and methanol [4]. Nonetheless, methanol, isoprene [31] and benzene [3, 30, 31] were associated with smoking itself. Nevertheless, the discrimination potential of Cyranose 320 to detect lung cancer was reported to be good [6, 7, 9].

Unfortunately, no international guidelines exist for sampling methodology for electronic nose analyses. Some of the collection-related factors, such as the effect of environmental volatile substances [32], humidity [33, 34] or sample storage [35] were addressed [24]. Alveolar gases may be influenced by breath hold (the period between inhalation and exhalation) and airway-borne substances are affected by expiratory flow rate [36]. In addition, in the investigation of lower airway molecules, the exclusion of anatomical dead space should also be standardised. It is not surprising that expiratory flow rate, the presence of breath hold and the inclusion of anatomic dead space may affect the levels of exhaled volatile compounds [33, 37, 38], however in electronic nose research, only the effect of expiratory flow rate has been investigated, but only in a small group of healthy subjects [14].

Therefore, the aim of this study was to analyse the effect of expiratory flow rate, breath hold and anatomic dead space on exhaled breath volatile compound pattern in lung cancer and health assessed with Cyranose 320.

## Methods

### Subjects

Thirty-seven healthy individuals (44 ± 14 years, mean ± SD) and 27 patients with lung cancer (60 ± 10 years) participated in the study. Twenty-seven healthy subjects were never-smokers and 10 were active smokers (31 ± 17 pack years). None of them have any chronic disorder, including asthma, COPD, allergy, diabetes, renal or hepatic disease. In smokers COPD has been excluded with lung function test.

All patients with lung cancer were current (n = 15) or ex-smokers (n = 12) with pack years of 60 ± 27. Lung cancer subjects were enrolled after the diagnosis has been established based on histology obtained either by endobronchial or transthoracic biopsy. Seventeen lung cancer patients had adenocarcinoma, 5 had squamous cell carcinoma, 3 had small cell carcinoma, 1 large cell carcinoma, 1 carcinoid and 1 sarcoma. Fourteen subjects were diagnosed newly and they did not receive any oncological treatment while 13 were receiving active chemotherapy. None of the lung cancer patients had asthma, allergy, renal of hepatic disorder, 12 patients were treated with COPD, and 4 with diabetes. All patients with COPD were using long-acting muscarin antagonists and 5 of them were on inhaled corticosteroids in combination with long-acting beta agonists.

None of the volunteers had respiratory tract infection 4 weeks prior to the study. All subjects were instructed to avoid consuming food and beverages [38] and not to perform physical exercise [39] at least for 2 hours before sample collection. Current smokers were asked to refrain from smoking for at least 6 hours prior to the measurements.

### Study design

All subjects were asked to perform four different breath collection procedures. Participants inhaled VOC-filtered room air with a deep inspiratory capacity manoeuvre then exhaled at a controlled flow rate (50 ml/sec) assessed with a flow-meter (VenThor, Thor Laboratories, Hungary) and, to close the soft palate, against resistance (15–20 cmH_2_O). The first 500 mL of exhaled air representing anatomic dead space was discarded using a small-resistance T-valve and the remaining air representing alveolar space was collected in a Teflon-coated Mylar bag. This method has originally been used for offline exhaled nitric oxide measurements (Ecomedics, Dürnten, Switzerland), but was standardised for VOC analyses by our workgroup and was shown to be reproducible within a day [40] and over 8 weeks [21]. The measurements with electronic nose were performed immediately after the collection. After attachment of a volatile molecule, sensors in Cyranose 320 respond with changes in resistance. After auto scale normalisation, sensor responses (dR) are calculated by the following formula: dR = (Rs-R)/R, where Rs is the response to the sampled gas and R is the response to the baseline reading, the reference gas being the VOC-filtered ambient room air.

To study the effect of expiratory flow rate, breath hold and dead space the previously described procedure was altered. Assessing the effect of expiratory flow rate subjects exhaled at 75 ml/sec which was compared to breath samples obtained at 50 ml/sec. To study the effect of breath hold, after deep inhalation through a VOC-filter, subjects held their breath at total lung capacity for 10 seconds and exhaled at 50 ml/sec. Finally, subjects performed an expiratory manoeuvre similar to the first one, but this time the dead space air was not discarded and mixed air was collected.

The study was approved by the University Ethics Committee (Semmelweis University, TUKEB 110/2007), and all subjects signed informed consent form prior to the measurements.

### Statistical analysis

SPSS 15.0 (SPSS Inc., Chicago, IL, USA), GraphPad Prism 5.03 (GraphPad Software Inc., San Diego, CA, USA) and Statistica 8.0 software (Stat Soft, Inc., Tulsa, OK, USA) were used for statistical analysis. To avoid the confounding effect of water vapour the four water sensitive sensors (sensors 5, 6, 23, 31) were excluded, thus in total 28 sensors were analysed. To reduce data dimensionality principal component analysis (PCA) was performed on sensor responses, principal components (PCs) were sorted based on their initial Eigen value sizes and the highest three (PC1, PC2 and PC3) were used for further analysis. To classify cases into categorical divisions, linear canonical discriminant analysis was used following a stepwise approach, where Mahalanobis-distance was applied to exclude outliers [41]. Leave-one-out cross validation was also performed with Mahalanobis distance followed by linear canonical discriminant analysis where the same dataset was used as the validation as well as the training groups. Pearson correlation, multivariate linear and logistic regression analyses were performed to detect relationships between clinical data and PCs.

The primary aim of this study was to investigate whether collection-related factors, including expiratory flow rate, breath hold and exclusion of anatomic dead space influence the breathprints of healthy subjects and patients with lung cancer. Repeated-measures ANOVA on principal components followed by the Dunnett’s post hoc test was applied to analyse this aim in two groups with 4 subsequent measurements. Principal components were derived from sensor responses, therefore *a priori* calculations on effect size were based on descriptive statistics of sensor responses in our previous cohorts [18, 40]. In these studies, the average standard deviation/mean ratio of the analysed (N = 28, after the exclusion of water-sensitive sensors) sensor responses was 0.35.

In addition, we showed that changes in exhalation flow rate and breath hold caused a 12% change in exhaled VOC levels [38]. Together these two (standard deviation/mean ratio of 0.35 and a change of 0.12) result an effect size of 0.35. Using an effect size of 0.35 and investigating two groups with 4 subsequent measurements and a power of 0.80 the minimal estimated sample size was N = 44 [42]. However, because of the considerable within group variability of the clinical data in patients with lung cancer, to improve the power of our results, we decided to exceed the minimally required sample size by enrolling 20 additional subjects.

Our secondary aim was to compare healthy and lung cancer groups when breath samples were collected with different collection setups. Post hoc power analysis based on a previous study [13] revealed that the standard error for correct classification between the healthy and lung cancer groups was below 6% for all comparisons. A p < 0.05 was considered significant.

## Results

### Effects of expiratory flow, breath hold and dead space on “breathprint”

**Figure 1:**
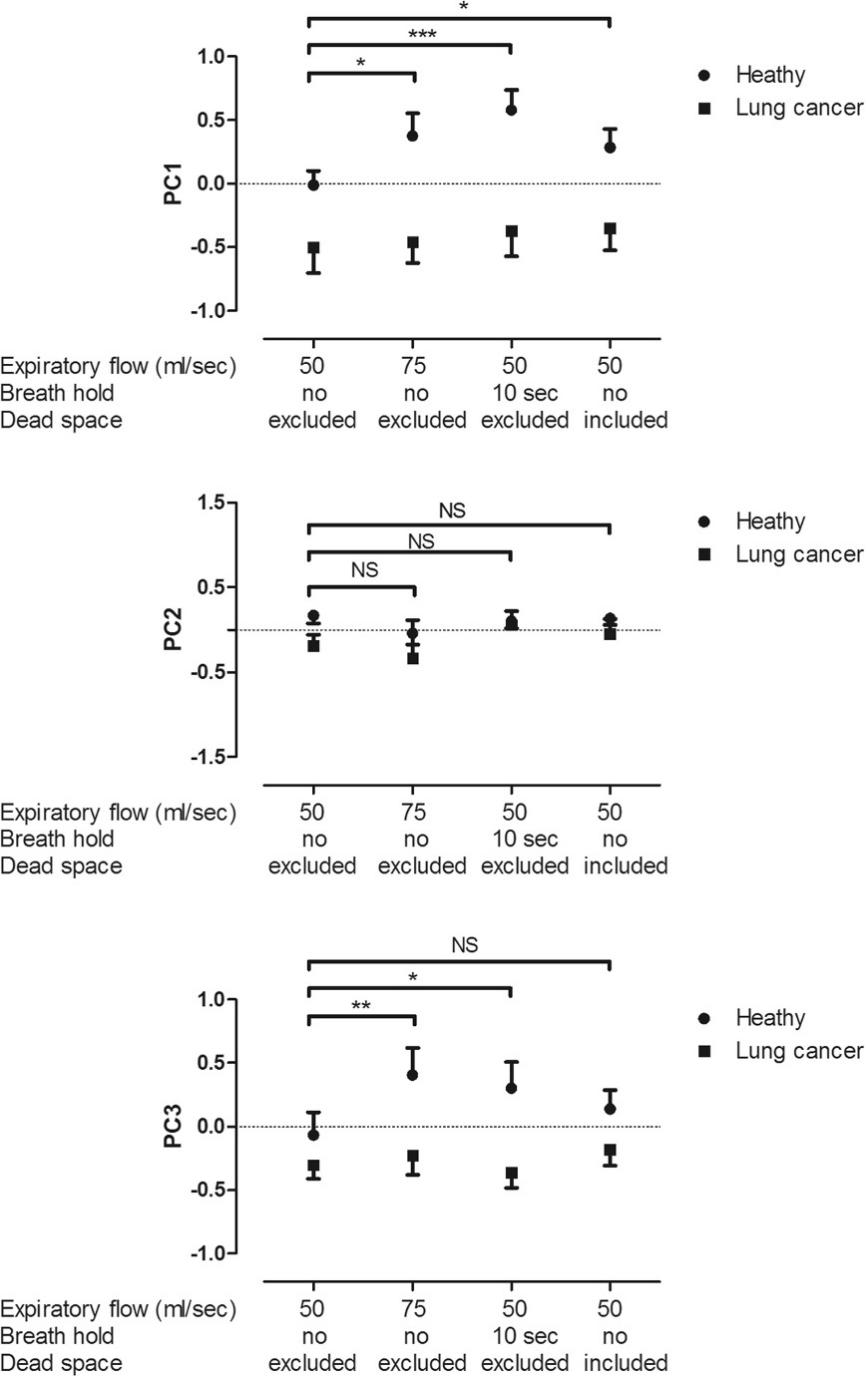
**The effect of expiratory flow, breath hold and dead space on principal components.** The collection-related factors caused significant differences only in healthy subjects (p < 0.05), the effect was not significant in patients with lung cancer (p > 0.05). Comparing to baseline measurements both higher expiratory flow and breath hold caused significant changes in PC1, PC2, PC3, while the inclusion of anatomic dead space affected only PC1. Data are expressed as mean ± SD. *-p < 0.05, **-p < 0.01, ***-p < 0.001, NS-not significant.

Expiratory flow rate, breath hold and dead space significantly altered exhaled “breathprints” only in healthy individuals (p < 0.05) but not in patients with lung cancer (p > 0.05, Figure 1).

Analysing individual sensor responses in healthy participants, expiratory flow rate affected all but sensors 2, 3, 7, 9, 19, 23, 27 and 32, breath hold changed all sensor responses, while dead space altered all but sensors 1, 12 and 13 significantly (p < 0.05). In patients with lung cancer, breath hold affected only sensor 29, while dead space altered responses of sensors 27 and 29 significantly (p < 0.05).

### The effect of sample collection on the ability of electronic nose to detect lung cancer

**Figure 2:**
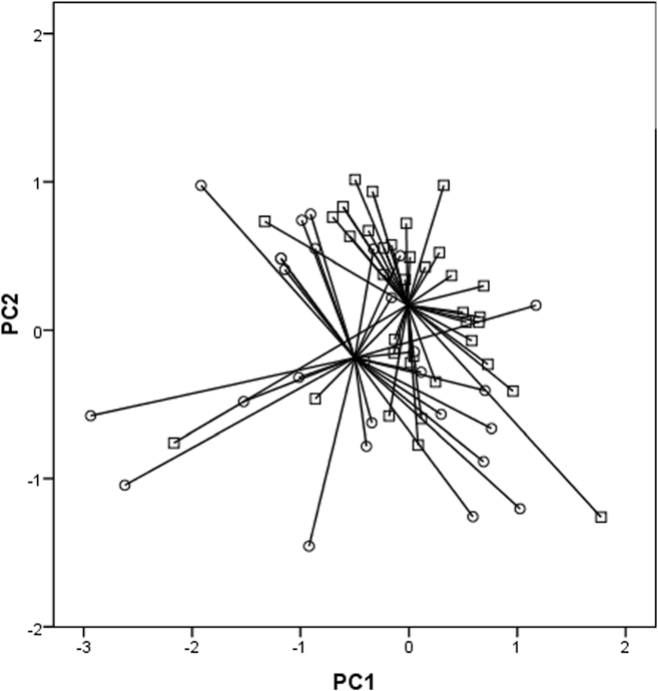
**Two dimensional PCA plot between healthy subjects (squares) and patients with lung cancer (circles).** Electronic nose could discriminate the two groups with a classification accuracy of 72% when breath samples were collected with a previously standardised collection procedure. The difference was significant (p = 0.02).

We found a significant difference between exhaled “breathprints” in healthy subjects and patients with lung cancer when the previously standardised collection setup (50 ml/sec, no breath hold and the exclusion of dead space) was used (p = 0.02, Figure 2). The electronic nose could discriminate the two groups with a classification accuracy of 72%, cross-validated accuracy of 70%, 63% sensitivity, 78% specificity, 63% positive predictive value and 78% negative predictive value. When only healthy smokers were compared to patients with lung cancer, the difference was still significant (p = 0.01, classification accuracy of 81%, 96% sensitivity, 40% specificity, 81% positive predictive value, 80% negative predictive value). Similarly, the difference was significant (p < 0.001) also when healthy never-smokers were compared to patients (classification accuracy of 74%, 67% sensitivity, 81% specificity, 78% positive predictive value, 71% negative predictive value).

When the healthy and lung cancer groups were compared at higher expiratory rate, the difference was significant with classification accuracy of 78%, cross-validated accuracy of 78%, 81% sensitivity, 76% specificity, 71% positive predictive value and 85% negative predictive value. The difference after 10 seconds of breath hold was also significant with classification accuracy of 70%, cross-validated accuracy of 70%, 78% sensitivity, 65% specificity, 62% positive predictive value and 80% negative predictive value. Although, the difference after inclusion of the dead space was still significant, the discrimination power was the poorest with classification accuracy of 70%, cross-validated accuracy of 69%, 67% sensitivity, 70% specificity, 64% positive predictive value and 75% negative predictive value.

### The effect of tumour histological type and active chemotherapeutic treatment on “breathprint”

When comparing various histological subtypes, no difference was observed in “breathprints” (p > 0.05). Similarly, there was no difference in “breathprints” between patients receiving active chemotherapy and treatment-naive patients (p > 0.05).

### The effects of lung function, age and smoking history on “breathprint”

**Figure 3:**
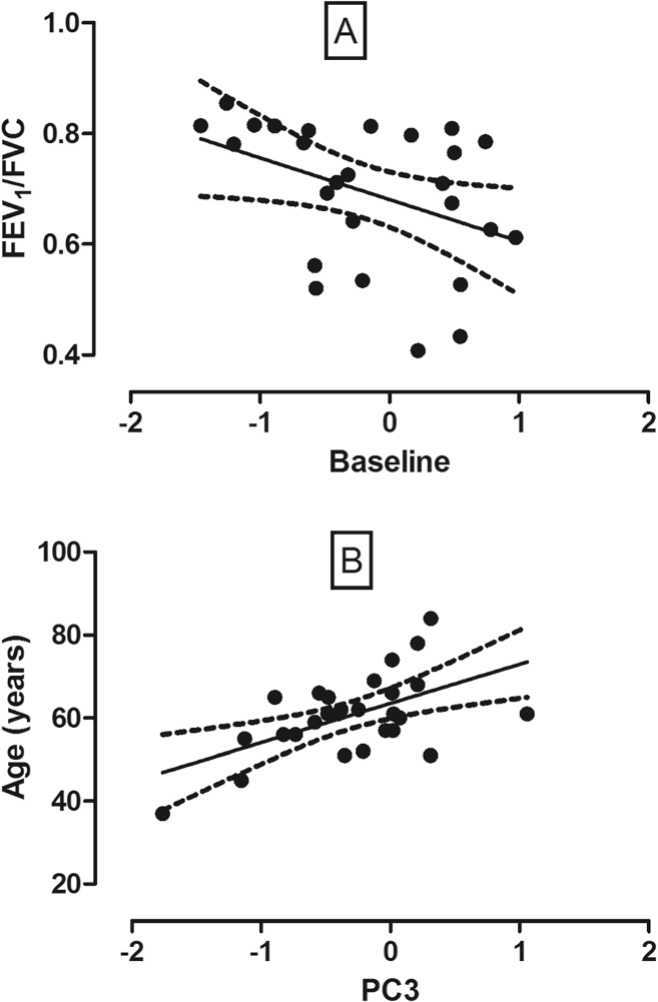
**Relationships between “breathprint” and FEV**_**1**_ **/FVC as well as age.** Significant associations were found between PC2 and FEV_1_/FVC (p = 0.03, r = 0.41, **Panel A**) as well as between PC3 and age (p = 0.002, r = 0.56, **Panel B**).

We found significant correlations between PC2 and FEV_1_ and PC2 and FEV_1_/FVC (r = 0.50 and r = 0.41, p < 0.05, Figure 3A) as well as PC3 and age (r = 0.56, p < 0.05, Figure 3B) in lung cancer subjects. Contrarily, there was no relationship between “breathprint” and lung function or “breathprint” and age in healthy subjects. Analysing individual sensors, we did not find significant relationship between sensor responses and lung function, age or smoking history in either group of subjects.

To exclude the potential confounding effect of COPD, comparison between healthy and lung cancer groups was also performed when only non-COPD lung cancer subjects were included, yielding still significant differences (p = 0.03, 60% sensitivity, 63% specificity). “Breathprints” of healthy smokers were significantly different from healthy never smokers (p = 0.01), while pack years were not related to “breathprints” in any group of subjects.

## Discussion

Electronic noses represent potential non-invasive tools for the early diagnosis of lung cancer. Unfortunately, there is still an unmet need for international guidelines for sampling methodology in electronic nose analyses. In this study we investigated collection-related methodological factors in an attempt to improve the standardisation of the sampling technique for electronic nose studies.

The expiratory flow rate might influence the breath level of molecules originating from the conducting airways. On one hand, the levels of volatile molecules with a steady axial diffusion, such as nitric oxide, may decrease at higher expiratory flow rate [36, 43]. On the other hand, if a VOC is taken up by the airway tissue, higher expiratory flow rate does not allow rapid diffusion towards the airway vessels and it is associated with elevated breath VOC levels [37]. Of note, if a molecule is released in the conducting airways it does not rule out the possibility that it also originates from the more distant part of the lungs. Flow-dependence of different VOCs has already been demonstrated [33, 37, 38, 44]. However, the airway kinetics of various volatile substances is not fully understood. It is known from experimental studies on nitric oxide that the relationship between expiratory flow rate and exhaled volatile compound levels may resemble a hyperbolic curve [43]. In line with this, proportionally higher expiratory flow rates may result only in marginal differences in exhaled volatile levels which might not be detected with electronic nose. Therefore, we decided to use two relatively slow expiratory flow rates in this study. In addition, these two relatively slow flow rates could have been achieved by all subjects, while our experience is that expiration with constant high flow-rates (>200 ml/sec) could be very struggling for very sick patients. Flow-dependency of exhaled volatile compound pattern has been investigated only in a preliminary experimental setup. In that study 100 to 200 ml/sec and 300 to 500 ml/sec expiratory flow rates were compared in 10 healthy non-smoker subjects without any significant difference [14]. However, the lack of difference may have been due to the relatively high target rates applied in that study. Of course, to understand the kinetics of VOC production in healthy and pathological airways more than two expiratory flow rates must be tested, and VOC levels should be measured with more precise instruments (i.e. GC-MS). The aim of the current study was only to demonstrate the importance of expiratory flow rate control.

In general, if subjects are not instructed properly, they may provide a breath sample after various times of breath hold [33]. The breath levels of molecules which are produced in the acinar airway/alveolar space or the ones which diffuse through the alveoli are influenced by breath hold. The longer the breath hold is, the more molecules accumulate in the airways resulting in higher breath levels [33, 36–38, 45].

The effect of dead space air on exhaled volatile compound concentrations is two-sided. Firstly, if a molecule is produced in the lower airways but not in the upper part, its concentrations may be diluted by the dead-space air. Secondly, some molecules may be produced in the upper airways influencing the results [46]. Therefore, comparison of alveolar (dead space excluded) and mixed air (dead space included) samples may reveal the origin of volatile molecules [27, 47]. Two possible approaches are introduced to avoid the problem of dead space; by partitioning the first proportion of exhaled air via a T-valve (time or volume-controlled separation) [46] or by monitoring the exhaled CO_2_ concentration and selecting the period when CO_2_ reaches the alveolar plateau (CO_2_-plateau controlled separation) [47]. A study comparing the two methods showed that the CO_2_-controlled method provides more precise results [27], however the time or volume-controlled separation which was used in the current study is simpler and more feasible.

It was shown that expiratory flow rate may alter acetone [44], ethanol [38], isoprene [33, 37] and pentane [37] levels, breath hold may increase isoprene [33, 37], methanol [44] and acetone [38, 44] concentrations and the inclusion of anatomic dead space may affect acetone levels [27]. Hence, it was not surprising that these factors altered “breathprints” significantly in the current study. Analysing the raw sensor data, collection-related factors altered different sensor responses suggesting that they may affect various volatile molecules differently. Interestingly, these effects were present only in healthy individuals. Various studied concluded decreased VOC levels in lung cancer compared to non-lung cancer control groups [2, 4]. This may imply that VOC levels in lung cancer were below the lower detection limit of electronic nose (0.1 ppm); therefore the lack of changes in lung cancer group was not due to physiological but analytical reasons. On the other hand, we have previously shown that expiratory flow dependency of VOCs may not be present in all subjects. Exhaled ethanol concentration in healthy volunteers with very low baseline levels was not affected by higher exhalation flow rate [38]. In addition, as some of the lung cancer subjects had also COPD, because of the possible airflow limitation, this might have been biased the kinetics of VOC production. This could contribute to the negative results in lung cancer patients.

Nevertheless, we found that collection-related factors influenced not only “breathprints” of healthy subjects but the differences between the two groups. Namely, the difference between healthy and lung cancer group widened when breath samples were obtained at higher expiratory flow rates. This further highlights the importance of sampling standardization, but also suggests possible opportunities to improve the classification accuracy of electronic nose. We used a leave-one out cross-validation method to test the discrimination ability of different collection setups. This technique has a certain limitation that the same dataset was used as the validation as well as the training groups. Therefore, the improved discrimination ability has to be tested blindly in future studies enrolling external group of new patients. Unfortunately, there is no consensus which is the best statistical approach for the classification of electronic nose data. A recent study highlighted that linear discriminant analysis, used also in the current study has a superior prediction accuracy, sensitivity and specificity in comparison to the other techniques, including partial least squares-discriminant analysis and random forests [48].

Theoretically, expiratory flow-, breath hold-, ant dead space-dependency of volatile substances may draw a conclusion about the anatomic site of production (i.e. conducting airways, alveoli or oral cavity, etc.). Electronic noses analyse a pattern of molecules, and it is plausible that volatile compounds contributing to “breathprint” originate from various sites. Unfortunately, the electronic nose used in this study is not able to separate exhaled volatile molecules; therefore, further studies coupling electronic noses with GC-MS are warranted to identify biomarkers associated with sampling-related changes.

Similarly to previous studies [6, 7] we found significant differences in “breathprint” between healthy subjects and patients with lung cancer. However these differences might not be related to the disease itself but to some other factors. For example, age and smoking history may alter exhaled molecules [13, 18, 24, 49]. We found a significant correlation between “breathprint” and age only in lung cancer subjects, and we cannot completely exclude the possibility that the difference in age between healthy and lung cancer groups might have biased our results. Of note, previous studies showed that age does not influence the discrimination ability between healthy controls and patients with obstructive airway diseases [13, 14]. Although smoking may change exhaled volatile substances [49], it was also shown that it did not affect the discrimination potential of exhaled volatile compound profile to detect lung cancer [7, 23]. Confirming the previous results [49], we also found a significant difference between active-smoker and never-smoker healthy subjects. Most notably, similarly to previous reports [7, 23], the difference between healthy volunteers and lung cancer patients was still present irrespectively whether the control group consisted of smokers or non-smokers. However, due to the potential effect of smoking, a comparison enrolling four groups of subjects (i.e. smoking healthy, non-smoking healthy, smoking patient and non-smoking patient) would be more accurate to exclude the potential effect of cigarette smoking. We found no difference in “breathprints” between active and ex-smoker lung cancer patients which was similar to the data found in COPD [12, 13]. The relationship between lung function and “breathprint” is not fully understood. We found a significant association between “breathprint” and lung function in lung cancer, however other authors showed no relationship between acute changes in airway calibre and exhaled volatile compound pattern [50]. The lack of relationship between “breathprint” and lung function in healthy subjects might be because of the relatively narrow range of lung function volumes in healthy individuals.

Important issues which are needed to be investigated in future studies include histological subtype of lung cancer, the role of chemotherapy and accompanying disorders. Studies examining exhaled breath showed differences in lung cancer compared to control subjects irrespectively from histology [5–7]. Some of our patients were on active oncologic treatment. Similarly to the previous study [5], electronic nose could classify lung cancer patients correctly disregarding their medical treatment history. In addition, there were no differences between the treated and untreated groups. However, this study has not been powered to analyse the effect of chemotherapy. COPD is frequently accompanied with lung cancer and it can modify exhaled “breathprint” itself [13]. Previous studies showed that exhaled “breathprint” differs in lung cancer when it was compared to COPD [5, 6]. Of note, in this study, the difference in lung cancer was still significant when non-COPD lung cancer patients were analysed. However, one may argue that in fact the results of the discrimination between lung cancer and controls will improve when a more homogeneous group is studied. To explore the potential of electronic nose in lung cancer screening further studies are warranted to understand possible confounding clinical factors.

## Conclusions

In summary, expiratory flow rate, breath hold and anatomic dead space may affect “breathprints” significantly. These effects may also influence the classification accuracy of electronic noses to separate disorders from health; by altering collection-related factors the discrimination ability may even be improved. Therefore, our study points to the need of methodological recommendations to standardise sample collection for electronic nose measurements.

## Abbreviations

ANOVA: Analysis of variances
COPD: Chronic obstructive pulmonary disease
FEV1: Forced expiratory volume in 1 second
FVC: Forced vital capacity
GC-MS: Gas-chromatography mass-spectrometry
PC: Principal component
PCA: Principal component analysis
VOC: Volatile organic compound.
